# Gold Nanoclusters Dispersed on Gold Dendrite-Based Carbon Fibre Microelectrodes for the Sensitive Detection of Nitric Oxide in Human Serum

**DOI:** 10.3390/bios12121128

**Published:** 2022-12-05

**Authors:** Mani Arivazhagan, Palanisamy Kannan, Govindhan Maduraiveeran

**Affiliations:** 1 Materials Electrochemistry Laboratory, Department of Chemistry, SRM Institute of Science and Technology, Kattankulathur, Chennai 603203, Tamil Nadu, India; 2 College of Biological, Chemical Sciences and Engineering, Jiaxing University, Jiaxing 314001, China

**Keywords:** carbon fibre, microelectrodes, gold nanoclusters, gold dendrites, electrochemical sensor, nitric oxide detection

## Abstract

Herein, gold nanoclusters (Au NC) dispersed on gold dendrite (Au DS)-based flexible carbon fibre (AuNC@AuDS|CF) microelectrodes are developed using a one-step electrochemical approach. The as-fabricated AuNC@AuDS|CF microelectrodes work as the prospective electrode materials for the sensitive detection of nitric oxide (NO) in a 0.1 M phosphate buffer (PB) solution. Carbon microfibre acts as an efficient matrix for the direct growth of AuNC@AuDS without any binder/extra reductant. The AuNC@AuDS|CF microelectrodes exhibit outstanding electrocatalytic activity towards NO oxidation, which is ascribed to their large electrochemical active surface area (ECSA), high electrical conductivity, and high dispersion of Au nanoclusters. As a result, the AuNC@AuDS|CF microelectrodes attain a rapid response time (3 s), a low limit of detection (LOD) (0.11 nM), high sensitivity (66.32 µA µM cm^−2^), a wide linear range (2 nM–7.7 µM), long-term stability, good reproducibility, and a strong anti-interference capability. Moreover, the present microsensor successfully tested for the discriminating detection of NO in real human serum samples, revealing its potential practicability.

## 1. Introduction 

Detection of nitric oxide (NO) is a critical to understanding cell functionality and pathology, as well as in diagnostic applications [1,2,3,4,5]. Nitric oxide is important not only in physiological processes, but also in pathophysiological practices [6,7,8,9,10]. It has further anti-inflammatory and immunosuppressive properties, which usually cause vasodilation and inhibit platelet adhesion, activation, and aggregation [11,12,13,14]. Changes in physiological NO concentration can lead to cardiovascular diseases such as hypertension, septicaemia, or atherosclerosis, as well as Parkinson’s disease or cancer [15,16,17,18,19]. Sensing NO is not only position-reliant, but also time- and concentration-reliant [20,21,22]. The detection of NO remains a difficult task because of its low-level concentration generated by cells, high reactivity, and short half-time [23,24,25]. Thus, the design and establishment of a reliable and high-performance NO sensor is key.

Among various analytical strategies, using electrochemical sensors and biosensors signifies one of the most capable approaches for sensing NO in clinical measurements due to their low limit of detection, prompt response, and ease of real sample analysis in low concentrations in biological samples [18,26,27,28,29]. In the development of NO sensing, there are countless benefits to the design of nanostructured sensing electrodes to improve the electron transfer process and achieve a high sensing performance [30,31,32]. Carbon fibre microelectrodes have been employed for various electrophysiological, electrochemical and biosensor systems owing to their relative chemical inertness and high mechanical and electrical characteristics [33,34,35,36]. A variety of carbon fibre-based sensor platforms were developed for the detection of catecholamines, glucose, NO, acetylcholine, choline, lactate, glutamate, etc. [37,38,39,40]. Furthermore, the small size of carbon fibre microelectrodes is effectively utilized for the miniaturization of sensor platforms. The dispersion or entrapment of various nanostructured materials such as graphene, carbon nanotubes, metal nanoparticles, metal oxides, etc., and DNA/enzymes onto carbon fibre microelectrodes has created new avenues for the sensitive electrochemical sensing of chemically and biologically important molecules [41,42,43].

Gold nanoclusters (AuNC) are small clusters (several to 100 gold atoms) that have appeared as emergent catalysts for various electrocatalytic reactions and biosensing applications because of their unique molecule-like characteristics, quantum confinement effects, high volume of active sites, and good biocompatibility [44,45]. The prompt development of Au nanocluster-based biosensor platforms has offered various opportunities in the field of clinical and biomedical applications [31,46,47]. Appropriate catalytic material-derived sensing electrodes can significantly enhance electrode kinetics, sensitivity and selectivity towards the detection of NO. Many Au or platinum (Pt) NC-based electrochemical sensors have widely reported numerous emergent biomarkers and metal ions [12,48,49,50], although the utilization of Au nanoclusters dispersed on carbon fibre microelectrodes has not demonstrated the sensitive detection of NO.

In this study, we report self-supported gold nanoclusters dispersed on gold dendrite-based flexible carbon fibre microelectrodes (AuNC@AuDS|CF) using a one-step electrochemical approach for the sensitive detection of NO. Owing to a large quantity of active sites and ECSA, high electrical conductivity, and high dispersion of Au nanoclusters on dendrite structures, the AuNC@AuDS|CF microelectrodes exhibit improved electrocatalytic activity and sensing performance towards the detection of NO. In addition, the AuNC@AuDS|CF microelectrode-based sensor platform exhibits a rapid response time, low limit of detection, high sensitivity, and strong anti-interference capability in practical human serum samples.

## 2. Experimental Section

### 2.1. Materials and Reagents

Gold (III) chloride trihydrate, sodium nitrite (NaNO_2_), lactic acid (LA), glucose (Glu), uric acid (UA), ascorbic acid (AA), paracetamol (PA), hydrogen peroxide (H_2_O_2_), monosodium dihydrogen phosphate dehydrate, and disodium hydrogen phosphate were received from Sigma Aldrich, St. Louis, MO, USA. Carbon fibre microelectrodes (diameter: ~300 μm) with a purity of 99.99% were purchased from Sigma Aldrich, St. Louis, MO, USA. Sulphuric acid (H_2_SO_4_) was obtained from Molychem, Mumbai, India. All reagents procured and applied in the present work were analytical-grade chemicals. Millipore Milli-Q water (resistivity ≥18 MΩ cm) was applied for the preparation of all solutions.

### 2.2. Fabrication of AuNC@AuDS|CF Microelectrodes

Gold nanoclusters dispersed on gold dendrites were directly grown on a carbon-fibre microelectrodes using a one-step electrochemical deposition method. Typically, carbon fibre microelectrodes (geometrical surface area of ~0.48 cm^2^ with radius of ~150 µm and height of ~5.0 mm) are cleaned through sonication in acetone followed by pure water. The carbon fibre microelectrodes were dipped in an electrolyte solution containing 5.0 mM HAuCl_4_ and 0.5 M H_2_SO_4_ and an applied constant potential of −0.2 V (vs Ag/AgCl) for 200 s [51]. The resulting AuNC@AuDS|CF microelectrodes were eroded with an abundant quantity of Millipore Milli-Q water at an ambient temperature and dried at 60 °C for 1 h.

### 2.3. Characterization of AuNC@AuDS|CF Microelectrodes

The as-developed AuNC@AuDS|CF microelectrodes were characterized via numerous physicochemical and electrochemical techniques. Primarily, a scanning electron microscopic (SEM) technique with Thermosceintific Apreo S and a transmission electron microscopic (TEM) technique with JEOL 2010F TEM were used for studying the surface morphology of the AuNC@AuDS|CF microelectrodes. The elemental existence and composition and their distribution on the AuNC@AuDS|CF microelectrodes were analysed with electron-dispersive X-ray (EDX) measurements with a Hitachi SU-70. X-ray diffraction (XRD) measurements were performed using a Pan analytical Xpert Pro Diffractometer. X-ray photoelectron spectroscopic (XPS) measurements were conducted for the AuNC@AuDS|CF microelectrodes using an XPS-PHI Versaprobe III.

All of the electroanalytical studies were conducted using the Electrochemical Origaflex multi-channel system (Origaflex OGF500) workstation at 26 ± 3 °C. The AuNC@AuDS|CF microelectrode was used as the working electrode, a platinum (Pt) wire was engaged as the auxiliary electrode, and an Ag/AgCl (3.0 M KCl) electrode acted as the reference electrode. All of the electrocatalytic and sensing measurements were performed in a 0.1 M phosphate buffer (PB, ~pH 2.5) electrolyte solution in an inert atmosphere. A chronoamperometric (CA) method was utilized for all the sensing measurements and the real sample analytical ability of the AuNC@AuDS|CF microelectrodes at the applied potential (*E*_app_) of ~0.82 V.

## 3. Results and Discussion

**Figure 1:**
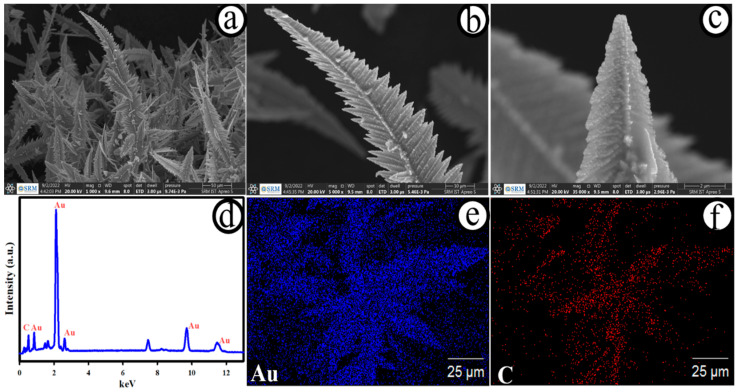
FE-SEM images (**a**–**c**), EDX spectrum (**d**) and elemental mapping (**e**,**f**) of the AuNC@AuDS|CF microelectrode.

Figure 1a–c presents the typical FE-SEM images of the developed AuNC@AuDS|CF microelectrode with low and high magnifications. As shown in Figure 1a, dendrite-like Au nanostructures were directly grown on carbon fibre microelectrodes with an average length of ~260 µm. A small dimension of Au nanoparticles with an average size of ~9 nm was homogeneously dispersed on Au dendrites (Figure 1b,c). Figure 1d depicts the energy-dispersive X-ray (EDX) spectra of the AuNC@AuDS|CF microelectrode, revealing the existence of carbon (C) and Au elements only on the electrode surface. The results of elemental mapping of Au and C are presented in Figure 1e,f, and the elements of Au were homogeneously distributed on the AuNC@AuDS|CF microelectrode. For the controlled study, the surface morphology of the carbon fibre electrode was analysed and is displayed in Figure S1, Supplementary Materials. The average dimension of the single carbon fibre was calculated to be ~5 μm, and the overall thickness of the carbon fibres was measured as ~300 μm, as represented in Figure S1a,b. After the deposition of AuNC@AuDS on carbon fibres, the geometrical surface area may be increased. The EDX (Figure S2, Supplementary Materials) and elemental mapping (Figure S3) study suggested that an element of C only existed on the carbon fibre microelectrodes and from environmental C existence, revealing the purity of the carbon fibres.

**Figure 2:**
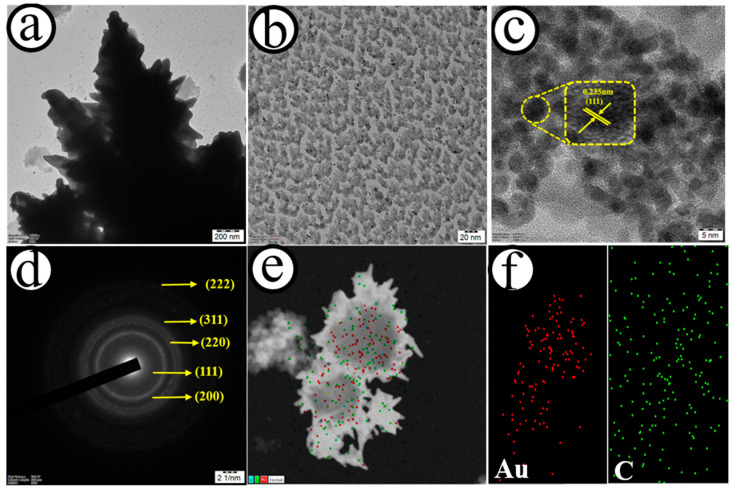
TEM (**a**,**b**), HRTEM (**c**), SAED pattern (**d**) and elemental mapping (**e**,**f**) of the AuNC@AuDS|CF microelectrode.

Figure 2 shows the transmission electron microscope (TEM) (a and b), high-resolution transmission electron microscope (HRTEM) (c), selected area electron diffraction (SAED) pattern (d), and elemental mapping (e and f) of the AuNC@AuDS|CF microelectrode. As depicted in Figure 2a,b, the TEM image of the AuNC@AuDS|CF microelectrode revealed that small-sized Au clusters, with a mean dimension of ~3.4 nm, were homogeneously distributed on Au dendrites. Based on Figure 2c, the value of lattice fringes was estimated as 0.235 nm, and corresponded to the crystalline plane of (111) Au [52]. The SAED pattern further confirmed the crystalline nature of the AuNC@AuDS|CF microelectrodes, showing a set of diffraction rings of the (111), (200), (220), (222) and (311) face-centred cubic structure of Au (Figure 2d). As depicted in Figure 2e,f and Figure S4, elements such as Au and C co-exist on the AuNC@AuDS|CF microelectrodes and were homogeneously presented.

**Figure 3:**
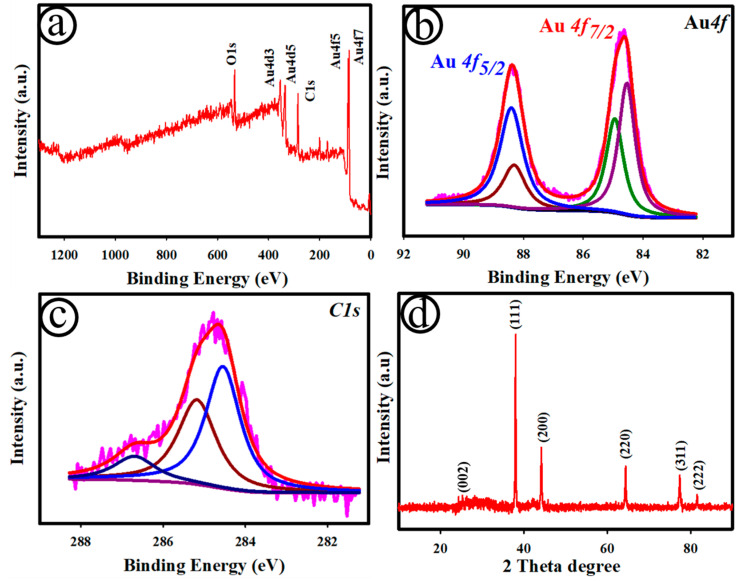
XPS survey spectra (**a**), and Au 4f (**b**) and C 1s (**c**) regions of the AuNC@AuDS|CF microelectrode; XRD pattern of the AuNC@AuDS|CF microelectrode (**d**).

Figure 3 depicts the XPS survey spectra (a), and the Au 4f (b) and C 1s (c) regions of the AuNC@AuDS|CF microelectrode. As displayed in Figure 3a, Au and C elements existed on the AuNC@AuDS|CF microelectrode. In the Au 4f region (Figure 3b), the XPS peaks appeared at ~84.61 eV (Au 4f_7/2_) and ~88.32 eV (Au 4f_5/2_), corresponding to the binding energies of Au^0^ [53]. Figure 3c displays the high-resolution XPS spectra for the C 1s region of the AuNC@AuDS|CF microelectrode. In Figure 3c, three major peaks were obtained: one centred at ~284.71 eV corresponding to C-C/C-H, and two at ~285.17 eV and ~286.78 eV, corresponding to the C=O and O-C=O groups, respectively, associated with the Au-C matrix. Figure 3d displays the XRD pattern of the AuNC@AuDS|CF microelectrode. The peaks acquired at the 2θ values of ~38.172, ~44.37, ~64.56, ~77.54 and ~81.7° were associated with the cubic crystalline nature of (111), (200), (220), (311) and (222) Au [52,54]. In addition, the XRD peak at ~25.6 can be assigned to the (002) plane of amorphous carbon, indicating that the electro-chemical deposition of Au NDS had not affected the crystallographic structure of the carbon fibres [55].

**Figure 4:**
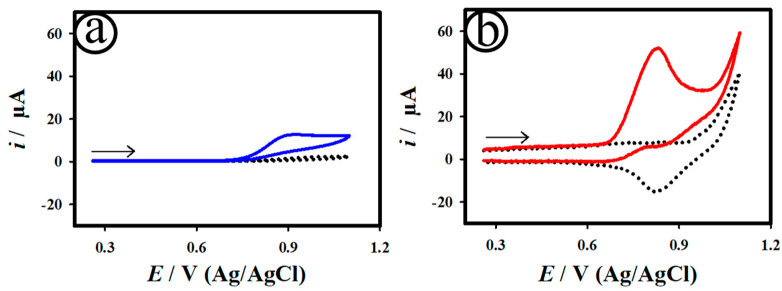
CV curves of the bare CF (**a**) and AuNC@AuDS|CF (**b**) microelectrodes measured in the nonexistence (dotted curve) and existence (solid curve) of 50.0 µM NO_2_^−^ in 0.1 M PB at a scan rate of 20 mVs^−1^.

Figure 4 shows the CV curves (a and b) of the bare CF and AuNC@AuDS|CF microelectrodes scanned in the nonexistence (dotted curve) and existence (solid curve) of 50.0 µM NO_2_^−^ in 0.1 M PB. As displayed in Figure 4a,b, the AuNC@AuDS|CF microelectrodes exhibited enhanced electrocatalytic oxidation of NO with an anodic current of ~0.043 mA at the potential of ~0.82 V after the addition of 50.0 µM NO_2_^−^. The perceived catalytic current was owing to the direct oxidation of NO at the AuNC@AuDS|CF microelectrodes (Equations (1)–(3)) [56]. However, the bare CF microelectrode exhibited a small catalytic anodic current of ~0.11 mA at ~0.9 V towards the NO oxidation. As anticipated, the direct electrochemical NO oxidation reaction on the AuNC@AuDS|CF microelectrodes is an irreversible process [57,58]. Figure S5a depicts the CV curves of the AuNC@AuDS|CF microelectrode recorded for 50.0 µM NO_2_^−^ in 0.1 M PB at different scan rates. The plot of anodic currents vs. the square root of scan rates for the AuNC@AuDS|CF microelectrode showed a linear plot (Figure S5b), revealing a diffusion-controlled process.

NO − e^−^ → NO^+^ (1)

NO^+^ + OH^−^ → NO_2_^−^ + H^+^ (2)

NO_2_^−^ + H_2_O → NO_3_^−^ + 2H^+^ + e^−^ (3)

To optimize the constant applied potential, the varied applied potentials of 0.6, 0.7 and 0.8 were applied on the bare CF and AuNC@AuDS|CF microelectrodes towards the amperometric detection of NO. The constant applied potentials were chosen based on the preliminary catalytic studies towards the oxidation of NO (Figure 4). As shown in Figure S6a,b, the AuNC@AuDS|CF microelectrodes exhibited the highest steady-state catalytic current of ~0.042 mA at the applied constant potential of ~0.8 V, whereas the bare CF microelectrodes offered less than ~0.01 mA. The as-developed AuNC@AuDS|CF microelectrodes revealed uppermost catalytic activity over ~4 times when compared to bare CF microelectrodes. In order to further understand the interfacial characteristics of the bare CF and AuNC@AuDS|CF microelectrodes, electrochemical impedance spectral (EIS) measurements were conducted for 50.0 µM NO_2_^−^ in 0.1 M PB, and the results are displayed in Figure S7. The fitted electronic equivalent circuit is represented in the inset of Figure S7. The Nyquist plot of the AuNC@AuDS|CF microelectrodes exhibited smaller polarization resistance (*R*_p_) of ~478 Ω cm^2^ and high capacitance value in comparison to the bare CF electrode (~854 Ω cm^2^). This result indicates that the AuNC@AuDS on CF microelectrodes facilitates electron transfer kinetics at the interface.

The accomplished high catalytic performance of the AuNC@AuDS|CF microelectrodes is due to the ascription of a densely formed small dimension of Au nanoclusters on the edges of the Au dendrites, offering a high quantity of active sites, ease of accessing NO, and intrinsic catalytic activity of Au. The electrochemical active surface area (ECSA) of the as-fabricated electrodes was calculated using the equation of *C*_dl_/*C*_s_, where *C*_dl_ represents double-layer capacitance and *C*_s_ represents the specific capacitance (~0.04 mF cm^−2^), both of which were measured via CV studies at different scan rates, starting from 10 to 125 mV s^−1^ (Figures S8 and S9). The value of the bare CF and AuNC@AuDS|CF microelectrodes was estimated to be ~0.034 and ~0.92 cm^2^, respectively. Thus, the as-developed AuNC@AuDS|CF exhibited extensive contact with the electrocatalytic active sites and extremely fascinating aptitude of NO at the electrode–electrolyte interface.

**Figure 5:**
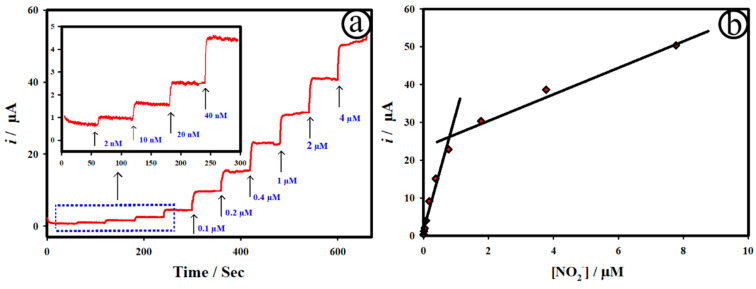
(**a**) Chronoamperometric *i*-t curve of the AuNC@AuDS|CF (**b**) microelectrodes upon adding various NO_2_^−^ concentrations (from 2 nM to 7.8 µM); (**b**) the calibration plot of Figure 5a.

Figure 5a displays the chronoamperometric *i*-t curve of the AuNC@AuDS|CF microelectrodes upon adding various concentrations of NO_2_^−^, starting from 2 nM to 7.8 µM in 0.1 M PB at the applied potential of 0.8 V (vs Ag/AgCl). The applied potential of 0.8 V for the electrochemical detection of NO_2_^−^ was chosen based on the good catalytic activity depicted in Figure 4. The anodic current increased rapidly and extended at a steady rate within ~3 s of the addition of NO_2_^−^ in 0.1 M PB. Figure 5b shows the calibration plot of Figure 5a, revealing a linear relationship to NO concentration. In short, two linear lines were attained for the AuNC@AuDS|CF microelectrodes upon adding various concentrations of NO_2_^−^, starting from 2.0 nm to 0.8 μM (correlation coefficient of *R*^2^ = 0.969) with a sensitivity of 66.32 μA μM^−1^ cm^−2^, and 1.8 μM to 7.7 μM (*R*^2^ = 0.991) with a sensitivity of 6.86 μA μM^−1^ cm^−2^. Owing to an increase in NO diffusion and high catalytic activity, the AuNC@AuDS|CF microelectrodes demonstrated high sensitivity (66.32 μA μM^−1^ cm^−2^) in the low concentration of NO. The catalytic activity of the AuNC@AuDS|CF microelectrodes may be affected by NO because of the adsorbed oxidized products at the electrode, delivering low sensitivity (6.86 μA μM^−1^ cm^−2^). Thus, the present sensor offered two linear lines towards the detection of NO. Moreover, the limit of detection was estimated as 0.11 nM through 3s/b, where “s” represents standard deviation of the blank and “b” means the slope. As shown in Table 1, the AuNC@AuDS|CF microelectrode-based sensor platform delivered results with the lowest detection limit, high sensitivity, and a wide linear range compared with recently reported NO sensors.

**Figure 6:**
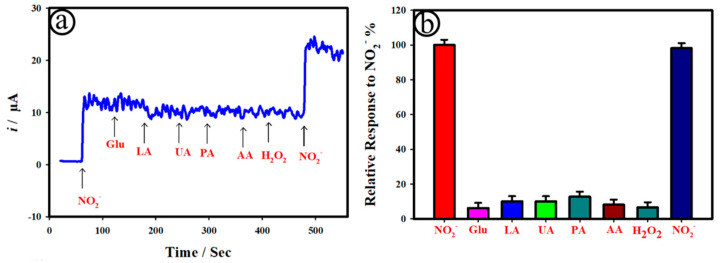
(**a**) *i*-t curve of the AuNC@AuDS|CF microelectrodes recorded upon addition of 100.0 nM NO_2_^−^, 1.0 µM glucose (Glu), 1.0 µM lactic acid (LA), 1.0 µM uric acid (UA), 1.0 µM paracetamol (PA), 1.0 µM ascorbic acid (AA), 1.0 µM H_2_O_2_, and 100.0 nM NO_2_^−^. *E*_app_ = 0.8 V. (**b**) The plot of the relative sensor response of AuNC@AuDS|CF microelectrodes to NO_2_^−^ in the existence of interferences.

Figure 6a shows the *i*-t curve of the AuNC@AuDS|CF microelectrodes recorded upon the addition of 100.0 nM NO_2_^−^ in the existence of electrochemically active interferences such as 1.0 µM glucose (Glu), 1.0 µM lactic acid (LA), 1.0 µM uric acid (UA), 1.0 µM paracetamol (PA), 1.0 µM ascorbic acid (AA), and 1.0 µM H_2_O_2_ at the applied potential (*E*_app_) of 0.8 V in 0.1 M PB. As can be seen in Figure 6a, the as-developed AuNC@AuDS|CF microelectrode responded with 100.0 nM NO_2_^−^ only. However, the AuNC@AuDS|CF microelectrode did not exhibit any catalytic activity towards 10-fold-high concentrations of electrochemically active interferences, including lactic acid, uric acid, paracetamol, ascorbic acid, and H_2_O_2_. Figure 6b presents the plot of the comparative sensing response of AuNC@AuDS|CF microelectrodes to NO_2_^−^ in the presence of other potential interferences. The obtained anodic current variation was measured to be ~5% towards the detection of NO for the AuNC@AuDS|CF microelectrode in the presence of interferences, revealing good selectivity.

**Figure 7:**
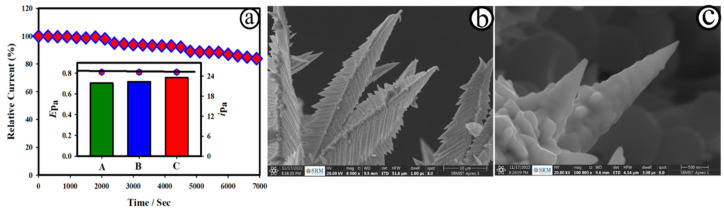
(**a**) Plot of relative catalytic activity of NO oxidation against time for the AuNC@AuDS|CF microelectrodes in 10 µM NO_2_^−^ + 0.1M PB for 7000 sec (*E*_app_: 0.8 V). (Inset) Plot of the catalytic activity of three brand-new AuNC@AuDS|CF microelectrodes recorded for 50 µM NO_2_^−^ 0.1M PB, derived from Figure S8. (**b**,**c**) FE-SEM images of AuNC@AuDS|CF microelectrodes after a stability test in real serum samples.

The durability of the as-developed AuNC@AuDS|CF microelectrode was tested in the presence of 10 µM NO_2_^−^ in 0.1M PB for 7000 sec at the applied potential of 0.8 V. Figure 7 shows the plot of relative catalytic activity of NO oxidation against time for the AuNC@AuDS|CF microelectrodes in 10 µM NO_2_^−^ + 0.1M PB. The AuNC@AuDS|CF microelectrode retained its catalytic activity (~88%) after 7000 sec of continuous activity, revealing the durability of the microelectrodes. The stability of the AuNC@AuDS|CF microelectrode was further tested in real human serum samples, and the results are shown in Figure S10. The catalytic activity of the as-developed AuNC@AuDS|CF microelectrode was reduced by ~16%, suggesting good durability, and retained dendrite structure (Figure 7b,c). The reproducible study was performed by measuring the CV curves of the three brand-new AuNC@AuDS|CF microelectrodes recorded for 50 µM NO_2_^−^ 0.1M PB at a scan rate of 20.0 mV s^−1^, as displayed in Figure S11. The inset of Figure 7 presents the plot of the catalytic activity of three brand-new AuNC@AuDS|CF microelectrodes (A–C) recorded for 50 µM NO_2_^−^ 0.1M PB, which were derived from Figure S11. The related standard deviation (RSD) was estimated to be 3.7% for three different AuNC@AuDS|CF microelectrodes, suggesting good reproducibility of the electrode.

Furthermore, the as-developed AuNC@AuDS|CF microelectrode-based sensor platform was tested with real human serum samples by employing the standard addition method (STM), and the results are summarized in Table 2. Interestingly, the AuNC@AuDS|CF microelectrodes exhibited recovery values in the range of 98.8–99.4% with an RSD range of 0.3–0.8 towards the detection of NO in human serum samples. The resulting microsensor demonstrated its potential practicability for the detection and determination of NO in real samples.

## 4. Conclusions

In the present study, self-supported gold nanoclusters (Au NC) dispersed on gold dendrite (Au DS)-based flexible carbon fibre (AuNC@AuDS|CF) microelectrodes (CFME) are established for sensing NO in human serum samples. A single-step electrochemical strategy was effectively adopted to fabricate AuNC@AuDS|CF microelectrodes where carbon microfibre acts as an efficient matrix for the direct growth of AuNC@AuDS without any binder/extra reductant. The AuNC@AuDS|CF microelectrodes serve as the emergent electrode materials for the enhanced electrocatalytic oxidation and sensitive detection of NO in 0.1 M PB because of its large quantity of ECSA, high electrical conductivity, and high dispersion of Au nanoclusters. The resulting AuNC@AuDS|CF microelectrodes delivered rapid response time of 3 s, a low limit of detection (LOD) of 0.11 nM, high sensitivity, a wide linear range of 2 nM–7.7 µM, long-term solidity, good reproducibility, and a strong anti-interference capability. The present microsensor also tested for the discriminating detection of NO in real human serum samples, revealing its potential practicability.

## Figures and Tables

**Table 1:** List of reported electrochemical sensors with present sensor based on AuNC@AuDS|CF microelectrodes towards the NO sensing.

Electrode	Technique	Sensitivity	Linear Range (μM)	LOD (nM)	Ref.
Au NPs/SG	Amperometry	45.44 μA mM⁻^1^ cm⁻^2^	10–2882	100 nM	[59]
NiFe–LDH NSAs/CC	Amperometry	3.46 µA cm^−2^mmol L^−1^	5–1000	20,000 nM	[57]
AuNPs/ERGO/GCE	Amperometry	5.38 µA μM^−1^ cm^−2^	25–200	133 nM	[60]
PEDOT-SH/Au/GCE	Amperometry	0.30 µA μM^−1^ cm^−2^	150–1000	51 nM	[61]
GNs/GC	Amperometry	6.32 µA μM^−1^ cm^−2^	0.5–45	220 nM	[62]
N-rGO	Amperometry	0.23 µA μM^−1^ cm^−2^	0.5–5000	200 nM	[63]
Pd-Cu-Mo_2_C/GCE	Amperometry	0.033 µA μM^−1^ cm^−2^	0.005–0.165	0.35 nM	[64]
Au NPs/MoS_2_/ GN/GCE	Amperometry	-	5–5000	1000 nM	[65]
AuNC@AuDS|CF	Amperometry	66.32 μA μM^−1^ cm⁻²	0.002–7.77	0.11 nM	This Work

Au NPs: gold nanoparticles; GCE: glassy carbon electrode; PEDOTpoly (3,4-ethylenedioxythiophene); NiFe-LDH NSAs: NiFe-layered double-hydroxide nanosheet arrays; CC: carbon cloth; NR: nano rods; rGO: reduced graphine oxide; MWCNTs: multi-walled carbon nanotubes; CF: carbon fibre; CeO_2_: cerium oxide; SnO_2_: tin oxide; ERGO: electrochemically reduced graphene oxide.

**Table 2:** Real sample analysis studied on the basis of recovery tests of NO_2_^−^ for the AuNC@AuDS|CF microelectrode for real human serum sample (n = 3).

Electrode	Added (nM)	Found ^a^ (nM)	Recovery (%)	RSD (%)
AuNC@AuDS|CF	10	9.92	99.20	0.31
	20	19.87	99.38	0.41
	50	49.38	98.76	0.76

^a^: Average of three measurements (n = 3).

## Data Availability

Not applicable.

## Supplementary Materials

The following are available online at https://www.mdpi.com/article/10.3390/bios12121128/s1: Figure S1. FE-SEM images of the bare carbon fibre microelectrode (a and b); Figure S2. EDX spectrum of the bare carbon-fibre microelectrode; Figure S3. Elemental mapping of the bare carbon-fibre microelectrode; Figure S4. EDX spectra for AuNC@AuDS|CF microelectrodes from TEM measurements; Figure S5. CV curves of the AuNC@AuDS|CF microelectrode recorded for 50.0 µM NO_2_^−^ in 0.1 M PB at different scan rates (a), The corresponding plot of anodic currents against the square root of scan rates (b); Figure S6. Chronoamperometric i-t curves of the bare CF (c) and AuNC@AuDS|CF (d) microelectrodes recorded in the nonexistence (dotted curve) and existence (solid curve) of 50.0 µM NO_2_^−^ in 0.1 M PB at different applied constant potentials; Figure S7. The Nyquist curves of the bare CF (a) and AuNC@AuDS|CF microelectrodes (b) recorded for 50.0 µM NO_2_^−^ in 0.1 M PB; Figure S8. (a) CV curves of the bare CF microelectrodes recorded in 0.1 M PB at different scan rates, starting from 10 to 125 mVs^−1^, (b) corresponding plot of anodic double layer currents against the scan rates; Figure S9. (a) CV curves of the AuNC@AuDS|CF microelectrodes recorded in 0.1 M PB at different scan rates, starting from 10 to 125 mVs^−1^, (b) corresponding plot of anodic double layer currents against the scan rates; Figure S10. Plot of relative catalytic activity of NO oxidation against time at the AuNC@AuDS|CF microelectrodes in 50 µM NO_2_^−^ + 0.1M PB in human serum samples for 5000 s (*E*_app_: 0.8 V); Figure S11. CV curves of the three brand-new AuNC@AuDS|CF microelectrodes recorded for 50 µM NO_2_^−^ 0.1M PB at a scan rate of 20.0 mV s^−1^.

## References

[1] Jae H.S., Privett B.J., Kita J.M., Wightman R.M., Schoenfisch M.H. (2008). Fluorinated Xerogel-Derived Microelectrodes for Amperometric Nitric Oxide Sensing. Anal. Chem..

[2] Hemmatian Z., Keene S., Josberger E., Miyake T., Arboleda C., Soto-Rodríguez J., Baneyx F., Rolandi M. (2016). Electronic Control of H+ Current in a Bioprotonic Device with Gramicidin A and Alamethicin. Nat. Commun..

[3] Manikandan V.S., Adhikari B.R., Chen A. (2018). Nanomaterial Based Electrochemical Sensors for the Safety and Quality Control of Food and Beverages. Analyst.

[4] Bamgboje D., Christoulakis I., Smanis I., Chavan G., Shah R., Malekzadeh M., Violaris I., Giannakeas N., Tsipouras M., Kalafatakis K. (2021). Continuous Non-Invasive Glucose Monitoring via Contact Lenses: Current Approaches and Future Perspectives. Biosensors.

[5] Sokolov A., Ali M., Li H., Jeon Y.R., Ko M.J., Choi C. (2021). Partially Oxidized MXene Ti3C2Tx Sheets for Memristor Having Synapse and Threshold Resistive Switching Characteristics. Adv. Electron. Mater..

[6] Deshpande A.S., Muraoka W., Andreescu S. (2021). Electrochemical Sensors for Oxidative Stress Monitoring. Curr. Opin. Electrochem..

[7] Li R., Qi H., Ma Y., Deng Y., Liu S., Jie Y., Jing J., He J., Zhang X., Wheatley L. (2020). A Flexible and Physically Transient Electrochemical Sensor for Real-Time Wireless Nitric Oxide Monitoring. Nat. Commun..

[8] Xu T., Scafa N., Xu L.P., Su L., Li C., Zhou S., Liu Y., Zhang X. (2014). Electrochemical Sensors for Nitric Oxide Detection in Biological Applications. Electroanalysis.

[9] Thirumalai D., Lee S., Kwon M., Paik H.J., Lee J., Chang S.C. (2021). Disposable Voltammetric Sensor Modified with Block Copolymer-Dispersed Graphene for Simultaneous Determination of Dopamine and Ascorbic Acid in Ex Vivo Mouse Brain Tissue. Biosensors.

[10] Mamdiwar S.D., Akshith R., Shakruwala Z., Chadha U., Srinivasan K., Chang C.Y. (2021). Recent Advances on Iot-Assisted Wearable Sensor Systems for Healthcare Monitoring. Biosensors.

[11] Russell C., Ward A.C., Vezza V., Hoskisson P., Alcorn D., Steenson D.P., Corrigan D.K. (2019). Development of a Needle Shaped Microelectrode for Electrochemical Detection of the Sepsis Biomarker Interleukin-6 (IL-6) in Real Time. Biosens. Bioelectron..

[12] Govindhan M., Chen A. (2016). Enhanced Electrochemical Sensing of Nitric Oxide Using a Nanocomposite Consisting of Platinum-Tungsten Nanoparticles, Reduced Graphene Oxide and an Ionic Liquid. Microchim. Acta.

[13] Dai Y., Song Y., Xie J., Xiao G., Li X., Li Z., Gao F., Zhang Y., He E., Xu S. (2020). CB1-Antibody Modified Liposomes for Targeted Modulation of Epileptiform Activities Synchronously Detected by Microelectrode Arrays. ACS Appl. Mater. Interfaces.

[14] Arivazhagan M., Santhosh Y.M., Maduraiveeran G. (2021). Non-enzymatic Glucose Detection Based on Nis Nanoclusters@nis Nanosphere in Human Serum and Urine. Micromachines.

[15] Maduraiveeran G., Sasidharan M., Jin W. (2019). Earth-Abundant Transition Metal and Metal Oxide Nanomaterials: Synthesis and Electrochemical Applications, Prog. Mater. Sci..

[16] He C., Tao M., Zhang C., He Y., Xu W., Liu Y., Zhu W. (2022). Microelectrode-Based Electrochemical Sensing Technology for in Vivo Detection of Dopamine: Recent Developments and Future Prospects. Crit. Rev. Anal. Chem..

[17] Wenninger N., Bračič U., Kollau A., Pungjunun K., Leitinger G., Kalcher K., Ortner A. (2021). Development of an Electrochemical Sensor for Nitric Oxide Based on Carbon Paste Electrode Modified with Nafion, Gold Nanoparticles and Graphene Nanoribbons. Sens. Actuators B Chem..

[18] Arivazhagan M., Shankar A., Maduraiveeran G. (2020). Hollow Sphere Nickel Sulfide Nanostructures–Based Enzyme Mimic Electrochemical Sensor Platform for Lactic Acid in Human Urine. Microchim. Acta.

[19] Goldoni R., Scolaro A., Boccalari E., Dolci C., Scarano A., Inchingolo F., Ravazzani P., Muti P., Tartaglia G. (2021). Malignancies and Biosensors: A Focus on Oral Cancer Detection through Salivary Biomarkers. Biosensors.

[20] Kannan P., John S.A. (2010). Highly Sensitive Electrochemical Determination of Nitric Oxide Using Fused Spherical Gold Nanoparticles Modified ITO Electrode. Electrochim. Acta.

[21] Dang X., Hu H., Wang S., Hu S. (2014). Nanomaterials-Based Electrochemical Sensors for Nitric Oxide. Microchim. Acta.

[22] Chaturvedi P., Vanegas D.C., Hauser B.A., Foster J.S., Sepúlveda M.S., McLamore E.S. (2017). Microprofiling Real Time Nitric Oxide Flux for Field Studies Using a Stratified Nanohybrid Carbon—Metal Electrode. Anal. Methods.

[23] Meenakshi S., Pandian K. (2016). Simultaneous Voltammetry Detection of Dopamine and Uric Acid in Pharmaceutical Products and Urine Samples Using Ferrocene Carboxylic Acid Primed Nanoclay Modified Glassy Carbon Electrode. J. Electrochem. Soc..

[24] Dong H., Zhou Y., Hao Y., Zhao L., Sun S., Zhang Y., Ye B., Xu M. (2020). “Turn-on” Ratiometric Electrochemical Detection of H2O2 in One Drop of Whole Blood Sample via a Novel Microelectrode Sensor. Biosens. Bioelectron..

[25] Yuan J., Jiang L., Che J., He G., Chen H. (2021). Composites of NiS2Microblocks, MoS2Nanosheets, and Reduced Graphene Oxide for Energy Storage and Electrochemical Detection of Bisphenol A. ACS Appl. Nano Mater..

[26] Arivazhagan M., Maduraiveeran G. (2020). Ultra-Fine Nickel Sulfide Nanoclusters @ Nickel Sulfide Microsphere as Enzyme-Free Electrode Materials for Sensitive Detection of Lactic Acid. J. Electroanal. Chem..

[27] Liu L., Zhang L., Dai Z., Tian Y. (2017). A Simple Functional Carbon Nanotube Fiber for: In Vivo Monitoring of NO in a Rat Brain Following Cerebral Ischemia. Analyst.

[28] Wo Y., Brisbois E.J., Bartlett R.H., Meyerhoff M.E. (2016). Recent Advances in Thromboresistant and Antimicrobial Polymers for Biomedical Applications: Just Say Yes to Nitric Oxide (NO). Biomater. Sci..

[29] Zuidema C., Schumacher C.S., Austin E., Carvlin G., Larson T.V., Spalt E.W., Zusman M., Gassett A.J., Seto E., Kaufman J.D. (2021). Deployment, Calibration, and Cross-Validation of Low-Cost Electrochemical Sensors for Carbon Monoxide, Nitrogen Oxides, and Ozone for an Epidemiological Study. Sensors.

[30] Maduraiveeran G., Jin W. (2017). Nanomaterials Based Electrochemical Sensor and Biosensor Platforms for Environmental Applications. Trends Environ. Anal. Chem..

[31] Arivazhagan M., Maduraiveeran G. (2022). Gold-Dispersed Hierarchical Flower-like Copper Oxide Microelectrodes for the Sensitive Detection of Glucose and Lactic Acid in Human Serum and Urine. Biomater. Sci..

[32] Maduraiveeran G. (2021). Nanoporous Structured Mixed Transition Metal Oxides Nanomaterials for Electrochemical Energy Conversion Technologies. Mater. Lett..

[33] Lawal A.T. (2019). Graphene-Based Nano Composites and Their Applications. A Review. Biosens. Bioelectron..

[34] Yu P., Wei H., Zhong P., Xue Y., Wu F., Liu Y., Fei J., Mao L. (2020). Single-Carbon-Fiber-Powered Microsensor for In Vivo Neurochemical Sensing with High Neuronal Compatibility. Angew. Chem.-Int. Ed..

[35] Chen Q., Mangadlao J.D., Wallat J., De Leon A., Pokorski J.K., Advincula R.C. (2017). 3D Printing Biocompatible Polyurethane/Poly(Lactic Acid)/Graphene Oxide Nanocomposites: Anisotropic Properties. ACS Appl. Mater. Interfaces.

[36] Bo X., Zhou M., Guo L. (2017). Electrochemical Sensors and Biosensors Based on Less Aggregated Graphene. Biosens. Bioelectron..

[37] Abdelbasir S.M., El-Sheikh S.M., Morgan V.L., Schmidt H., Casso-Hartmann L.M., Vanegas D.C., Velez-Torres I., McLamore E.S. (2018). Graphene-Anchored Cuprous Oxide Nanoparticles from Waste Electric Cables for Electrochemical Sensing. ACS Sustain. Chem. Eng..

[38] Wongkaew N., Simsek M., Griesche C., Baeumner A.J. (2019). Functional Nanomaterials and Nanostructures Enhancing Electrochemical Biosensors and Lab-on-a-Chip Performances: Recent Progress, Applications, and Future Perspective. Chem. Rev..

[39] Durairaj S., Sidhureddy B., Cirone J., Chen A. (2018). Nanomaterials-Based Electrochemical Sensors for In Vitro and In Vivo Analyses of Neurotransmitters. Appl. Sci..

[40] Jiang D., Zhang Q., Xu C., Ge Y., Huang L., Ren X., Wang Y. (2020). Facile Preparation of a Hollow Core-Shell Nanocomposite for the Ultrasensitive Sensing of Glucose. Sens. Actuators B Chem..

[41] Ramachandran R., Chen T.-W., Chen S.-M., Baskar T., Kannan R., Elumalai P., Raja P., Jeyapragasam T., Dinakaran K., Kumar G.P.G. (2019). A Review of the Advanced Developments of Electrochemical Sensors for the Detection of Toxic and Bioactive Molecules. Inorg. Chem. Front..

[42] Liu H., Weng L., Yang C. (2017). A Review on Nanomaterial-Based Electrochemical Sensors for H2O2, H2S and NO inside Cells or Released by Cells. Microchim. Acta.

[43] Xu C., Wu F., Yu P., Mao L. (2019). In Vivo Electrochemical Sensors for Neurochemicals: Recent Update. ACS Sens..

[44] Verma S., Singh A., Shukla A., Kaswan J., Arora K., Ramirez-Vick J., Singh P., Singh S.P. (2017). Anti-IL8/AuNPs-RGO/ITO as an Immunosensing Platform for Noninvasive Electrochemical Detection of Oral Cancer. ACS Appl. Mater. Interfaces.

[45] Sweet C., Pramanik A., Jones S., Ray P.C. (2017). Two-Photon Fluorescent Molybdenum Disulfide Dots for Targeted Prostate Cancer Imaging in the Biological II Window. ACS Omega.

[46] Maduraiveeran G., Ramaraj R. (2017). Gold Nanoparticle-Based Sensing Platform of Hydrazine, Sulfite, and Nitrite for Food Safety and Environmental Monitoring. J. Anal. Sci. Technol..

[47] Cheng X., Li Y., Kou J., Liao D., Zhang W., Yin L., Man S., Ma L. (2022). Novel Non-Nucleic Acid Targets Detection Strategies Based on CRISPR/Cas Toolboxes: A Review. Biosens. Bioelectron..

[48] Sehit E., Altintas Z. (2020). Significance of Nanomaterials in Electrochemical Glucose Sensors: An Updated Review (2016–2020). Biosens. Bioelectron..

[49] Li Y., Zhang Y., Li F., Feng J., Li M., Chen L., Dong Y. (2017). Ultrasensitive Electrochemical Immunosensor for Quantitative Detection of SCCA Using Co_3_O_4_@CeO_2_-Au@Pt Nanocomposite as Enzyme-Mimetic Labels. Biosens. Bioelectron..

[50] Reghunath R., Devi K., Singh K.K. (2021). Recent Advances in Graphene Based Electrochemical Glucose Sensor. Nano-Struct. Nano-Objects.

[51] Thangavel S., Ramaraj R. (2008). Polymer Membrane Stabilized Gold Nanostructures Modified Electrode and Its Application in Nitric Oxide Detection. J. Phys. Chem. C.

[52] Kim B., Song W.C., Park S.Y., Park G. (2021). Green Synthesis of Silver and Gold Nanoparticles via Sargassum Serratifolium Extract for Catalytic Reduction of Organic Dyes. Catalysts.

[53] Nazemi M., Soule L., Liu M., El-Sayed M.A. (2020). Ambient Ammonia Electrosynthesis from Nitrogen and Water by Incorporating Palladium in Bimetallic Gold–Silver Nanocages. J. Electrochem. Soc..

[54] Krishnamurthy S., Esterle A., Sharma N.C., Sahi S.V. (2014). Yucca-Derived Synthesis of Gold Nanomaterial and Their Catalytic Potential. Nanoscale Res. Lett..

[55] Sekar M., Pandiaraj M., Bhansali S., Ponpandian N., Viswanathan C. (2019). Carbon Fiber Based Electrochemical Sensor for Sweat Cortisol Measurement. Sci. Rep..

[56] Klyamer D., Shutilov R., Basova T. (2022). Recent Advances in Phthalocyanine and Porphyrin-Based Materials as Active Layers for Nitric Oxide Chemical Sensors. Sensors.

[57] Ma Y., Wang Y., Xie D., Gu Y., Zhang H., Wang G., Zhang Y., Zhao H., Wong P.K. (2018). NiFe-Layered Double Hydroxide Nanosheet Arrays Supported on Carbon Cloth for Highly Sensitive Detection of Nitrite. ACS Appl. Mater. Interfaces.

[58] Zhe T., Li R., Wang Q., Shi D., Li F., Liu Y., Liang S., Sun X., Cao Y., Wang L. (2020). In Situ Preparation of FeSe Nanorods-Functionalized Carbon Cloth for Efficient and Stable Electrochemical Detection of Nitrite. Sens. Actuators B Chem..

[59] Lu L. (2019). Highly Sensitive Detection of Nitrite at a Novel Electrochemical Sensor Based on Mutually Stabilized Pt Nanoclusters Doped CoO Nanohybrid. Sens. Actuators B Chem..

[60] Ting S.L., Guo C.X., Leong K.C., Kim D.H., Li C.M., Chen P. (2013). Gold Nanoparticles Decorated Reduced Graphene Oxide for Detecting the Presence and Cellular Release of Nitric Oxide. Electrochim. Acta.

[61] Ge Y., Jamal R., Zhang R., Zhang W., Yu Z., Yan Y., Liu Y., Abdiryim T. (2020). Electrochemical Synthesis of Multilayered PEDOT/PEDOT-SH/Au Nanocomposites for Electrochemical Sensing of Nitrite. Microchim. Acta.

[62] Mehmeti E., Stanković D.M., Hajrizi A., Kalcher K. (2016). The Use of Graphene Nanoribbons as Efficient Electrochemical Sensing Material for Nitrite Determination. Talanta.

[63] Chen D., Jiang J., Du X. (2016). Electrocatalytic Oxidation of Nitrite Using Metal-Free Nitrogen-Doped Reduced Graphene Oxide Nanosheets for Sensitive Detection. Talanta.

[64] Vilian A.T.E., Umapathi R., Hwang S.K., Huh Y.S., Han Y.K. (2021). Pd–Cu Nanospheres Supported on Mo2C for the Electrochemical Sensing of Nitrites. J. Hazard. Mater..

[65] Han Y., Zhang R., Dong C., Cheng F., Guo Y. (2019). Sensitive Electrochemical Sensor for Nitrite Ions Based on Rose-like AuNPs/MoS2/Graphene Composite. Biosens. Bioelectron..

