# Supplementary material for: Gold Nanoclusters Dispersed on Gold Dendrite-Based Carbon Fibre Microelectrodes for the Sensitive Detection of Nitric Oxide in Human Serum
Source: Biosensors (Basel). 2022 Dec 5;12(12):1128. doi: 10.3390/bios12121128 (PMC9776376; doi:10.3390/bios12121128)
Supplement: Supplementary file 1 [file biosensors-12-01128-s001.zip › biosensors-2033954-supplementary.pdf]

biosensors

Supporting Information

Gold Nanoclusters Dispersed on Gold Dendrites based Carbon-Fibres Microelectrodes for the Sensitive Detection of Nitric Oxide in Human Serum

Mani Arivazhagan and Govindhan Maduraiveeran*

Materials Electrochemistry Laboratory, Department of Chemistry, SRM Institute of Science and Technology, Kattankulathur 603 203, Tamil Nadu, India

**Corresponding Author E-mail: maduraig@srmist.edu.in*

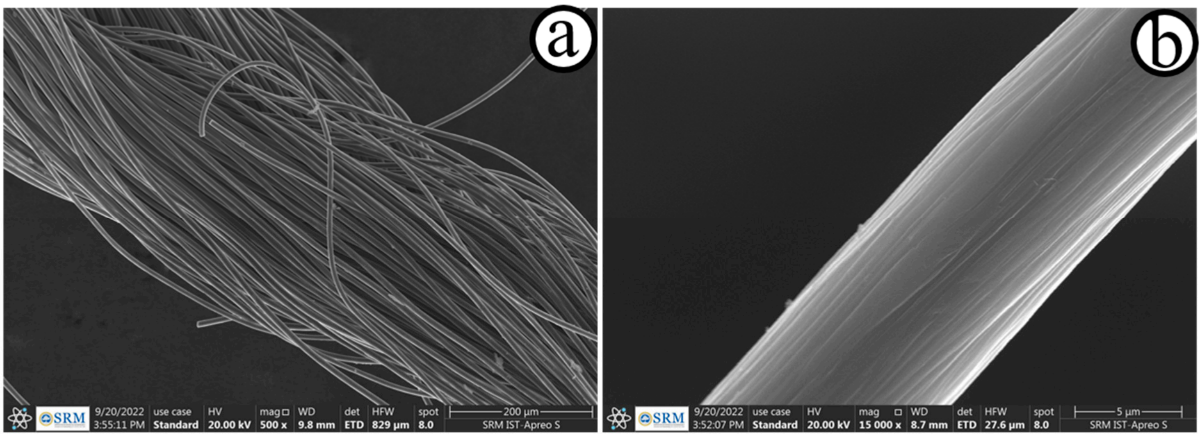

Figure S1. FE-SEM images of the bare carbon-fibres microelectrode (a & b).

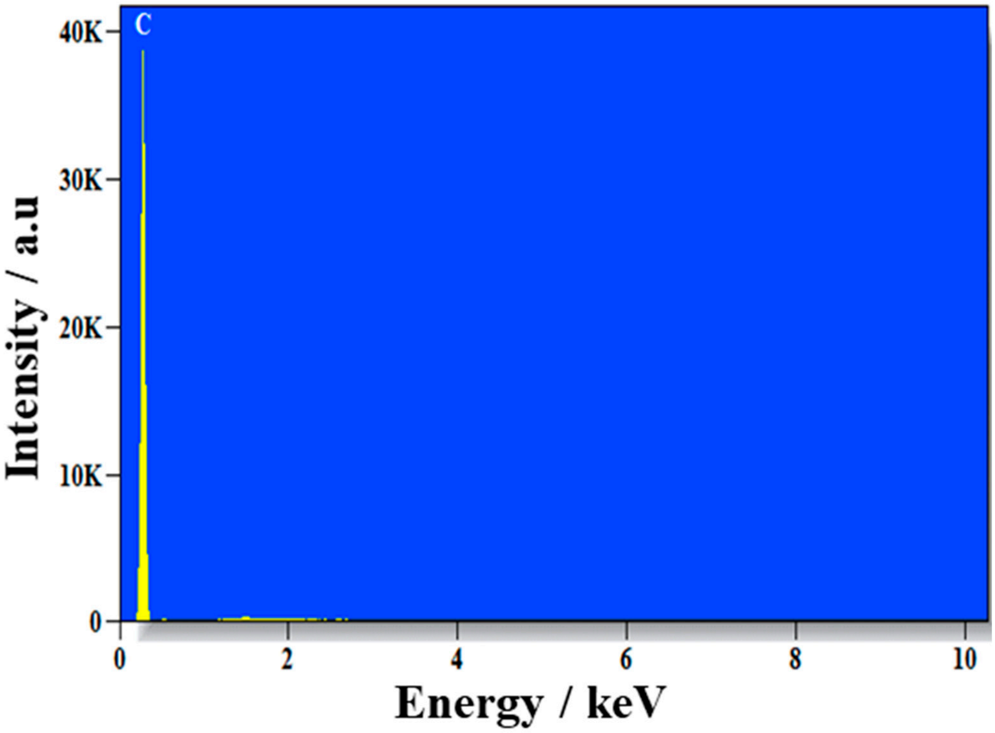

Figure S2. EDX spectrum of the bare carbon-fibres microelectrode.

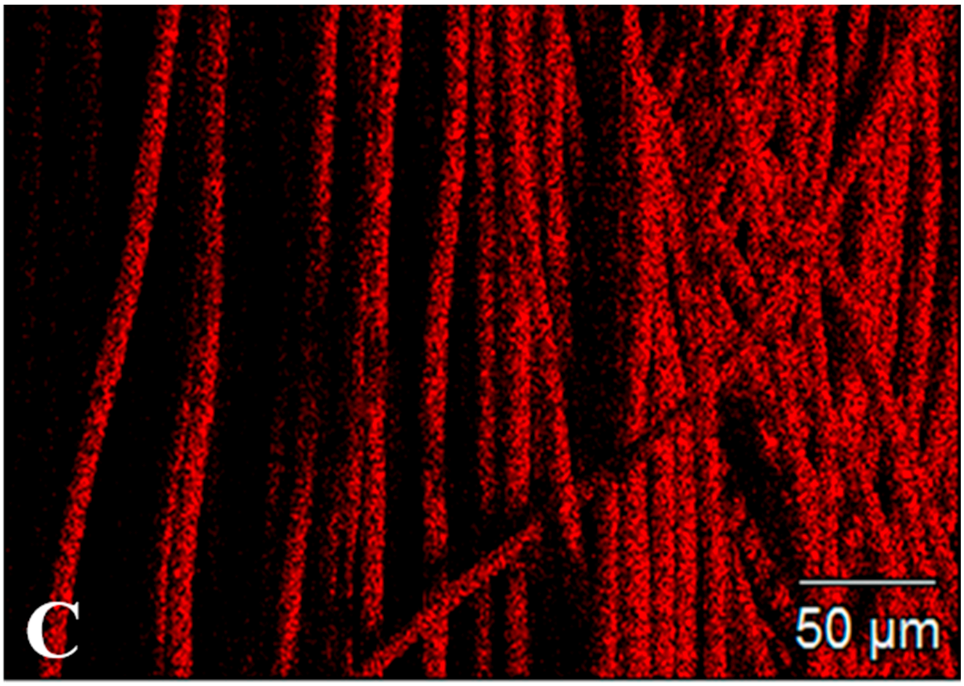

Figure S3. Elemental mapping of the bare carbon-fibres microelectrode.

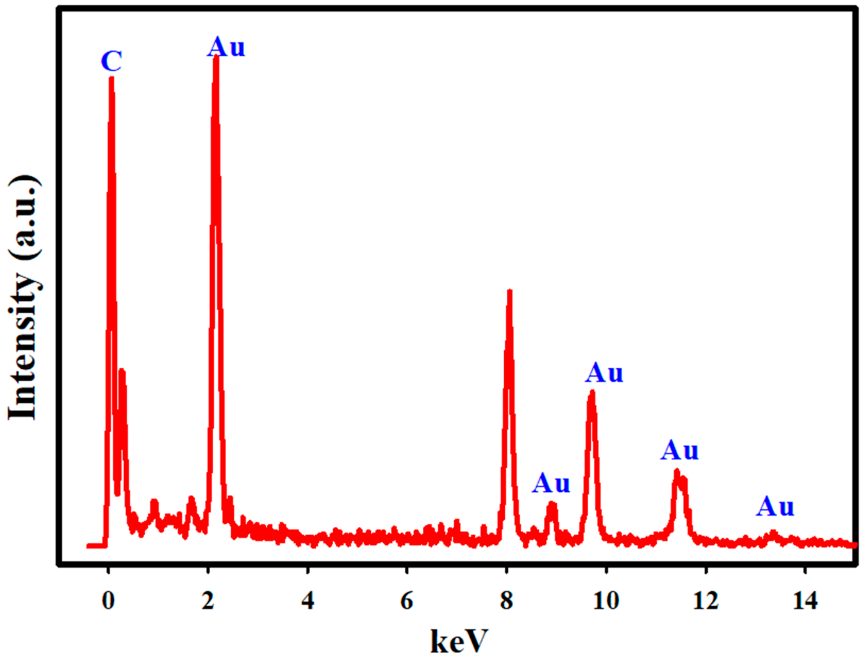

Figure S4. EDX spectra for AuNC@AuDS|CF microelectrodes from TEM measurements.

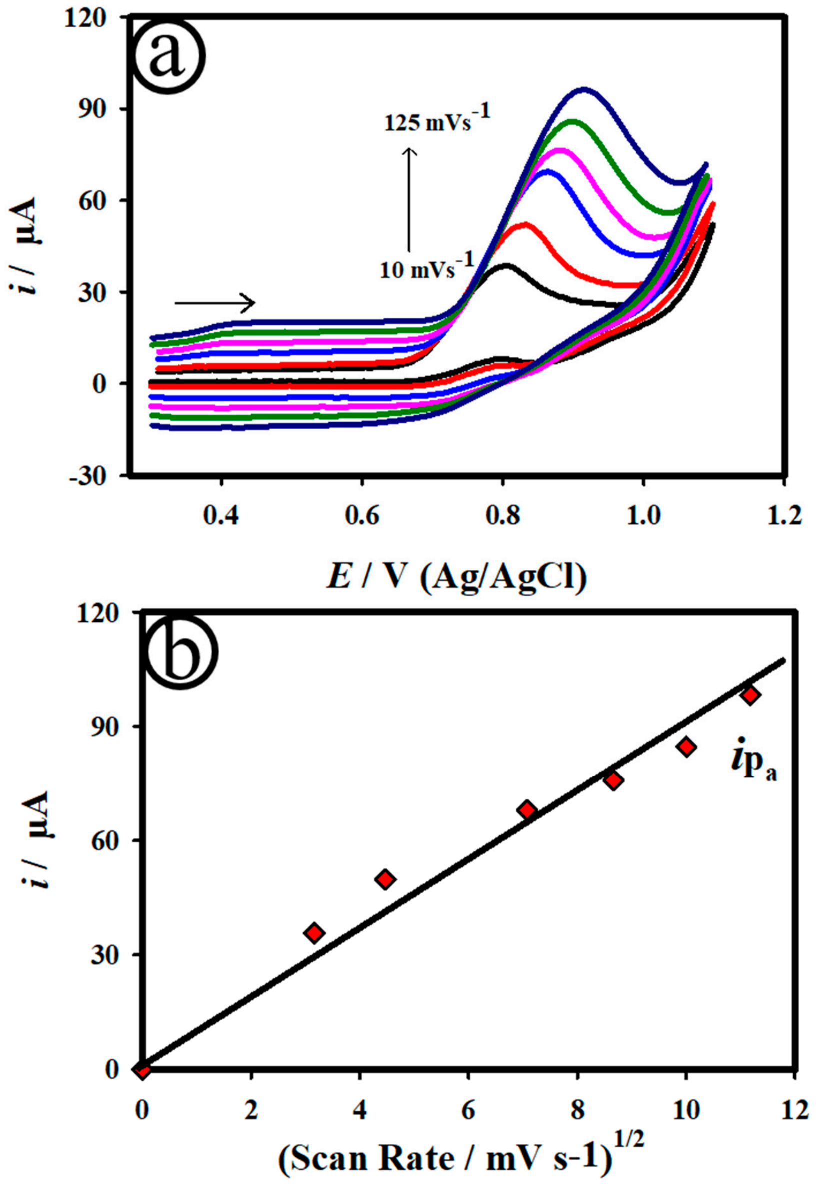

Figure S5. CV curves of the AuNC@AuDS|CF microelectrode recorded for 50.0 µM NO_2^- in 0.1 M PB at different scan rates (a). The corresponding plot of anodic currents against the square root of scan rates (b).

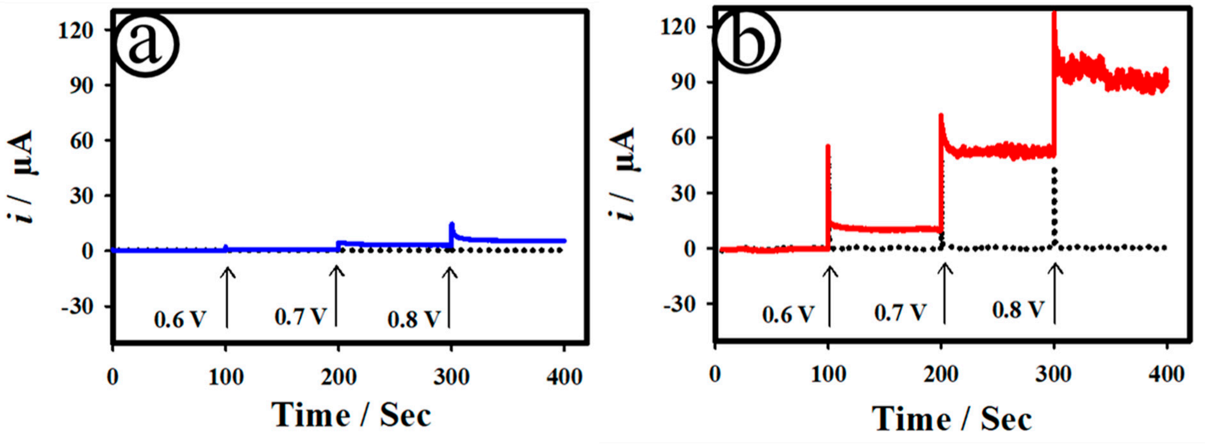

Figure S6. Chronoamperometric *i*-*t* curves of the bare CF (c) and AuNC@AuDS|CF (d) microelectrodes recorded in the nonexistence (dotted curve) and existence (solid curve) of 50.0 µM NO_2^- in 0.1 M PB at different applied constant potentials.

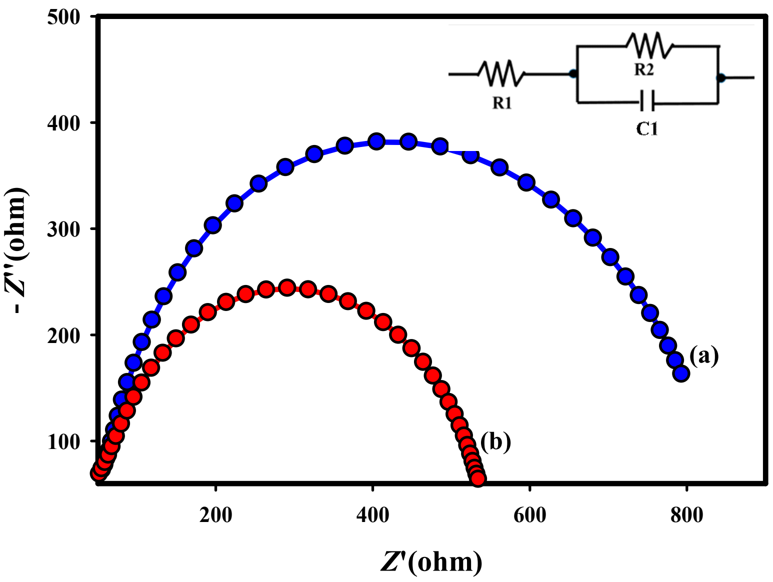

Figure S7. The Nyquist curves of the bare CF (a) and AuNC@AuDS|CF microelectrodes (b) recorded for 50.0 μM NO_2^- in 0.1 M PB.

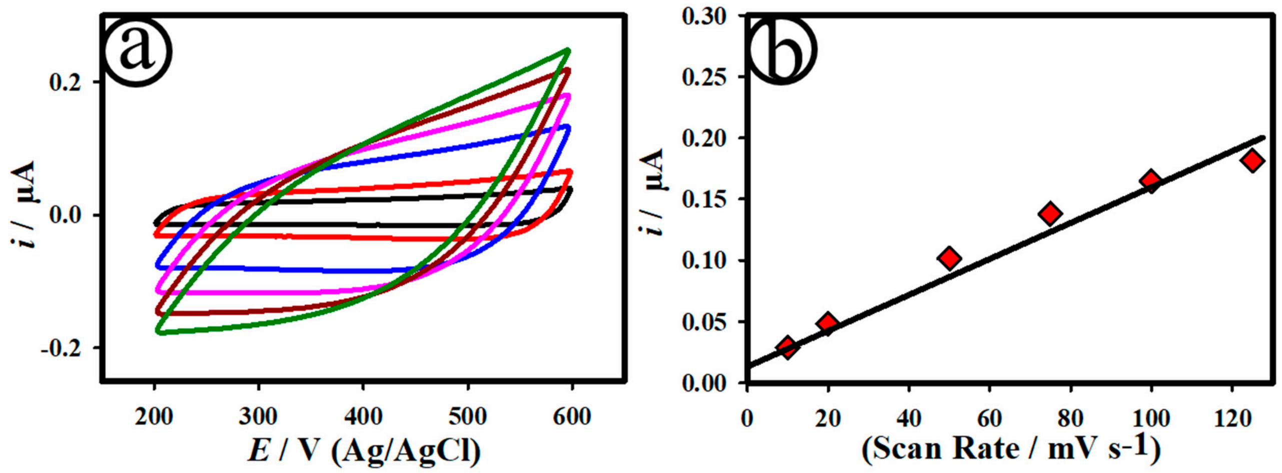

Figure S8. (a) CV curves of the bare CF microelectrodes recorded in 0.1 M PB at different scan rates, starting from 10 to 125 mVs^{-1}. (b) Corresponding plot of anodic double layer currents against the scan rates.

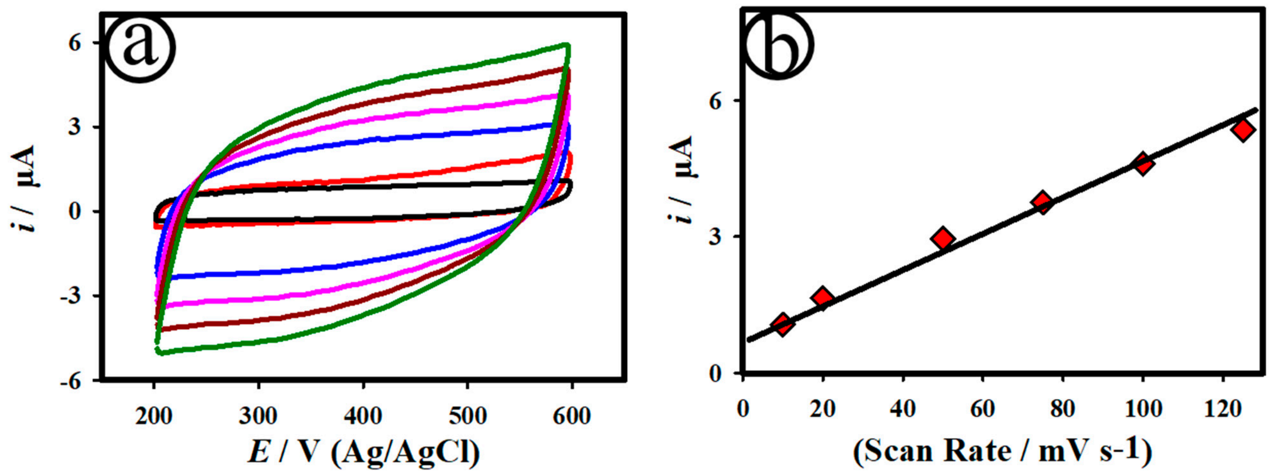

Figure S9. (a) CV curves of the AuNC@AuDS|CF microelectrodes recorded in 0.1 M PB at different scan rates, starting from 10 to 125 mVs^{-1}. (b) Corresponding plot of anodic double layer currents against the scan rates.

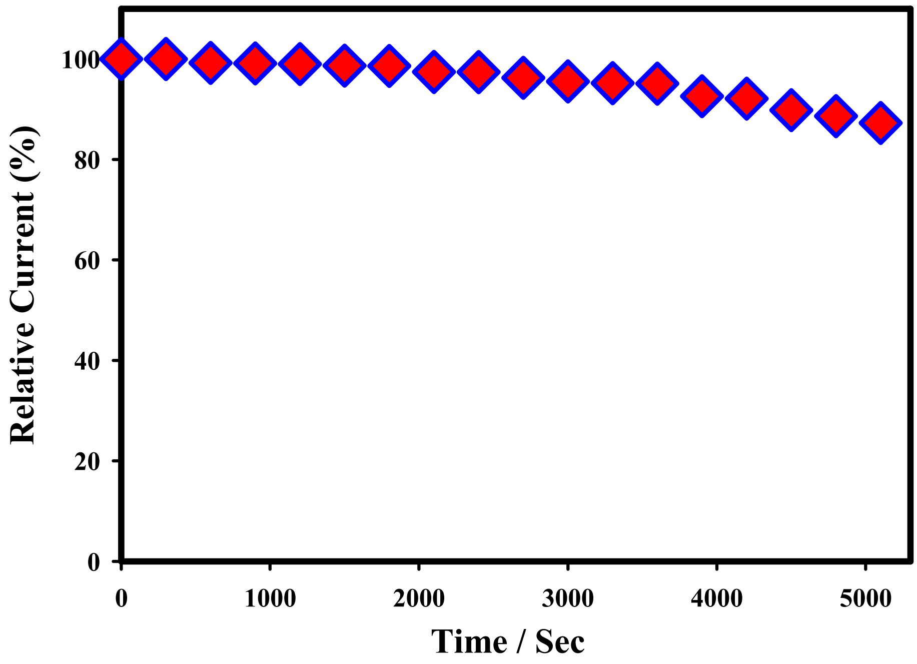

Figure S10. Plot of relative catalytic of NO oxidation against time at the AuNC@AuDS|CF microelectrodes in 50 µM NO_2^- + 0.1M PB in human serum samples for 5000 sec (E_{app}: 0.8 V).

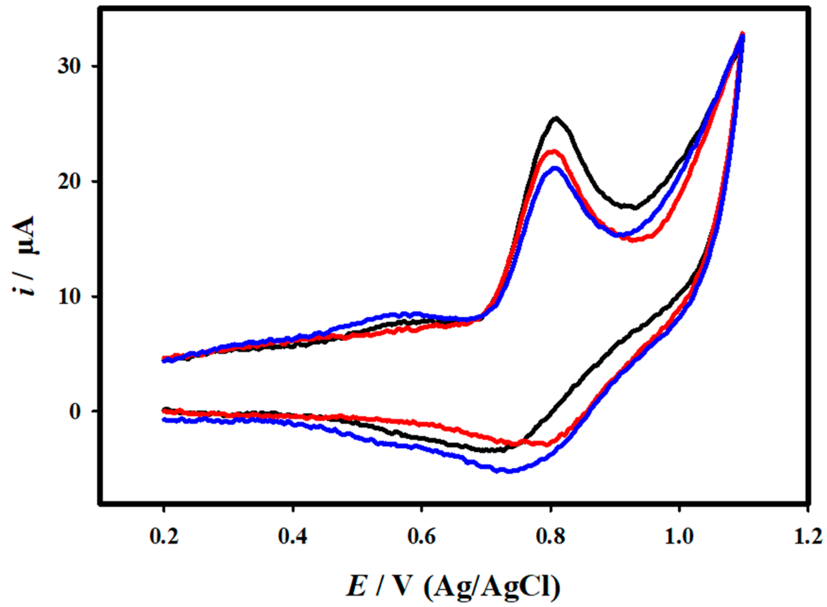

Figure S11. CV curves of the three-brand new AuNC@AuDS|CF microelectrodes recorded for 50 µM NO_2^- 0.1M PB at a scan rate of 20.0 mV s^{-1}.
